# RIP4 inhibits STAT3 signaling to sustain lung adenocarcinoma differentiation

**DOI:** 10.1038/cdd.2017.81

**Published:** 2017-06-02

**Authors:** Jawahar Kopparam, Johanna Chiffelle, Paolo Angelino, Alessandra Piersigilli, Nadine Zangger, Mauro Delorenzi, Etienne Meylan

**Affiliations:** 1 Swiss Institute for Experimental Cancer Research, School of Life Sciences, Ecole Polytechnique Fédérale de Lausanne, Lausanne CH-1015, Switzerland; 2 Bioinformatics Core Facility, Swiss Institute of Bioinformatics, Lausanne CH-1015, Switzerland; 3 Institute of Animal Pathology, University of Bern, Länggassstrasse 122, Bern CH-3012, Switzerland; 4 Histology Core Facility, School of Life Sciences, Ecole Polytechnique Fédérale de Lausanne, Lausanne CH-1015, Switzerland; 5 Ludwig Center for Cancer Research, University of Lausanne, Epalinges CH-1066, Switzerland; 6 Department of Oncology, Faculty of Biology and Medicine, University of Lausanne, Lausanne CH-1011, Switzerland

## Abstract

Loss of epithelial differentiation and extracellular matrix (ECM) remodeling are known to facilitate cancer progression and are associated with poor prognosis in patients with lung cancer. We have identified Receptor-interacting serine/threonine protein kinase 4 (RIP4) as a regulator of tumor differentiation in lung adenocarcinoma (AC). Bioinformatics analyses of human lung AC samples showed that poorly differentiated tumors express low levels of RIP4, whereas high levels are associated with better overall survival. *In vitro*, lung tumor cells expressing reduced RIP4 levels showed enhanced activation of STAT3 signaling and had a greater ability to invade through collagen. In contrast, overexpression of RIP4 inhibited STAT3 activation, which abrogated interleukin-6-dependent induction of lysyl oxidase, a collagen cross-linking enzyme. In an autochthonous mouse model of lung AC initiated by Kras(G12D) expression with loss of p53, Rip4 knockdown tumors progressed to a poorly differentiated state marked by an increase in Hmga2, reduced Ttf1, and enrichment of genes regulating extracellular remodeling and Jak-Stat signaling. Tail vein injections of cells overexpressing Rip4 showed a reduced potential to invade and form tumors, which was restored by co-expression of Stat3. Altogether, our work has identified that loss of RIP4 enhances STAT3 signaling in lung cancer cells, promoting the expression of ECM remodeling genes and cancer dedifferentiation.

Dedifferentiation of adenocarcinoma (AC) has been linked to various features of cancer development such as invasion, metastasis and stemness of cancer cells.^1, 2^ Mechanisms involved in dedifferentiation of lung tumors, the leading cause of cancer-related death,^3^ are poorly understood. Nuclear factor-kappa B (NF-*κ*B) and Signal transducer and activator of transcription 3 (STAT3) are two transcription factors often activated in lung AC,^4, 5, 6^ and promote dedifferentiation.^7, 8^ Tissue inhibitor of metalloproteinases 1 (TIMP1), a NF-*κ*B target gene coding for an enzyme involved in extracellular matrix (ECM) catabolism, was shown to promote cellular proliferation in lung AC and is associated with a poor prognosis in patients.^9^ Interleukin-6 (IL6), a target of NF-*κ*B and a major activator of STAT3 signaling, promotes pleural effusion and metastasis of lung cancer.^10, 11^ Recent studies in lung AC with mutant oncogenic Kirsten rat sarcoma virus (KRAS), found in 30% of the human disease,^12^ have shown that Stat3 acts as a tumor suppressor during cancer initiation but promotes cancer progression and metastasis in established tumors in an Il6-dependent manner.^13, 14, 15^ In multiple tumor types, STAT3 is known to regulate ECM remodeling enzymes such as matrix metalloproteinases and collagen deposition.^16, 17, 18^ These studies suggest that STAT3 signaling is activated in different steps of tumor progression including dedifferentiation and metastasis.

Receptor-interacting serine/threonine protein kinases (RIP) are known to impact cell fate decisions by regulating NF-*κ*B and Tumor necrosis factor (TNF) signaling amongst others.^19, 20^ Upon TNF stimulation, RIP1 mediates NF-*κ*B activation, in the absence of which cells undergo apoptosis.^21^ RIP3 mediated phosphorylation of RIP1 was shown to activate a pronecrotic complex downstream of TNF stimulation.^22^ Upon bacterial infection, RIP2 was shown to activate NF-*κ*B in a Nucleotide-binding oligomerization domain-dependent manner.^23^ Recent *in vitro* studies have hinted towards a role for RIP members in cancer invasion and metastasis. Indeed, treatment of lung cancer cells with TNF-related apoptosis-inducing ligand led to RIP1-mediated STAT3 activation resulting in increased cell invasion;^24^ RIP2-mediated activation of NF-*κ*B was crucial for triple-negative breast cancer cell motility and invasion.^25^

RIP4, an ankyrin repeat-containing kinase, promotes keratinocyte differentiation and inhibits cell migration during wound healing.^26^ *Rip4* knockout mice are characterized by poorly differentiated and dysplastic epidermis.^27^ In humans, mutations in *RIP4* have been linked to an autosomal-recessive disorder called Bartsocas-Papas syndrome, which is characterized by loss of epidermal differentiation.^28, 29^ In cancer, RIP4 has been found to have conflicting roles: in diffuse large B-cell lymphoma (DLBCL) RIP4 promotes cancer progression by activating the NF-*κ*B pathway and protects the cancer cells from chemotherapy-mediated cell death.^30^ In contrast, retroviral insertional mutagenesis in hepatocytes identified RIP4 as a tumor suppressor in hepatocellular carcinoma.^31^ These studies suggest that RIP4 has a context specific function in cancer progression and in regulating cellular differentiation.

To investigate the role of RIP4 in lung AC progression, we employed an inducible autochthonous mouse model where tumors are initiated upon conditional expression of oncogenic *KrasG12D* and loss of tumor suppressor *p53* (referred to as KP mice from here on).^32^ This model develops lung AC that recapitulates many aspects of the human disease. A previous study reported that Il6 signaling was activated in this model in advanced lung AC.^33^ In our study, we show that RIP4 inhibits STAT3 signaling and lung cancer dedifferentiation.

## Results

### Rip4 sustains lung AC differentiation

**Figure 1:**
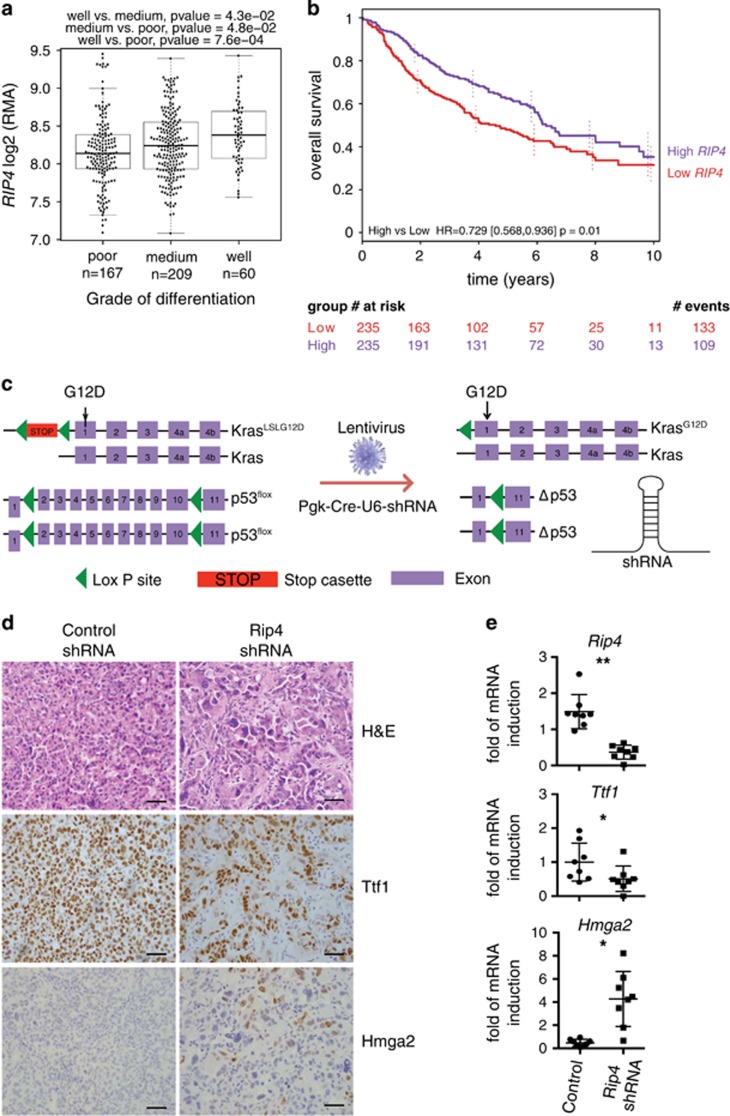
Rip4 regulates lung AC differentiation. (**a**) *RIP4* mRNA levels in human lung AC samples were plotted against grade of differentiation. (**b**) Overall survival of patients with lung AC, divided into two groups based on median *RIP4* expression (high and low), is represented as a Kaplan–Meier plot. Number of patients who have a follow-up for each year and group has been indicated at the bottom. Cox proportional hazards regression model, *P*=0.01, HR=0.729. (**c**) Schematic of the mouse model of lung AC is shown. *Kras*^*LSLG12D/WT*^*; p53*^*fl/fl*^ mice were intra-tracheally infected with lentivirus carrying cre recombinase and shRNA of interest (Rip4 or control). (**d**) Tumors were examined 18.5 weeks after tumor initiation. Representative images of paraffin-embedded lung tumor sections with control shRNA (left) or Rip4 shRNA (right) were stained with H&E, Ttf1 and Hmga2. Scale bars represent 20 *μ*m. (**e**) Real-time PCR was performed on cDNA generated from tumor mRNA using probes for *Rip4*, *Ttf1* or *Hmga2*. Data show mean±S.D. (*n*=8) Mann–Whitney test was used. *P<0.05, **P<0.01. AC, adenocarcinoma; HR, hazard ratio; RMA, robust multi-array average

As a first approach, we investigated the expression of *RIP4* in a compiled publicly available data set of human lung AC samples. *RIP4* mRNA levels were significantly higher in well-differentiated tumors compared to medium or poorly differentiated tumors (Figure 1a). Interestingly, higher *RIP4* expression is associated with better overall survival (Figure 1b) and, from The Cancer Genome Atlas (TCGA) data set, with *TP53* but not *KRAS* mutation status (Supplementary Figures S1a and b). We then analyzed the effect of Rip4 on tumor progression in an autochthonous lung AC model. Tumors were initiated by expression of *Kras*^*G12D*^ from its endogenous locus, with loss of *p53* (KP) and expression of a stable shRNA in lung tumor cells throughout tumor development (Figure 1c). Haematoxylin & Eosin (H&E) staining of control tumors eighteen weeks later showed well-differentiated papillary and glandular structures. In marked contrast, tumors with Rip4 knockdown had an increased frequency of undifferentiated patterns accompanied by multinucleated giant neoplastic cells (Figure 1d). Analysis of *Rip4* mRNA levels by real-time PCR confirmed sustained Rip4 knockdown in tumors with shRip4 (Figure 1e). Tumor volumes, as measured by micro-computed tomography (*μ*CT) prior sacrifice, were not different upon Rip4 knockdown (Supplementary Figure S1c). Immunohistochemistry (IHC) staining for phospho-histone H3 (pHH3) and cleaved caspase 3 (CC3) revealed no difference in proliferation or apoptosis (Supplementary Figure S1d). To confirm the observed altered differentiation at the molecular level, we analyzed the levels of Ttf1 and Hmga2 by IHC and real-time PCR. Ttf1 is a marker of well-differentiated lung AC, which is reduced upon dedifferentiation and metastasis.^34^ In contrast, Hmga2 is highly expressed in poorly differentiated and aggressive lung AC.^35^ In tumors with Rip4 knockdown, there was a higher Hmga2 and a reduced Ttf1 expression, confirming the loss of differentiation of these tumors (Figures 1d and e,Supplementary Figure S1e). As Rip4 is known to alter the NF-*κ*B pathway, we investigated NF-*κ*B activity. IHC revealed no noticeable difference in nuclear staining of the NF-*κ*B p65 subunit in Rip4 knockdown compared to control tumors (Supplementary Figure S1d). In addition, knockdown of RIP4 using siRNA in the human NCI-H2009 lung AC cell line showed no difference in nuclear p65, p100 or p52 levels (Supplementary Figure S1f). Hence, Rip4 knockdown leads to lung tumor dedifferentiation without affecting NF-*κ*B.

### RIP4 inhibits STAT3 activation

**Figure 2:**
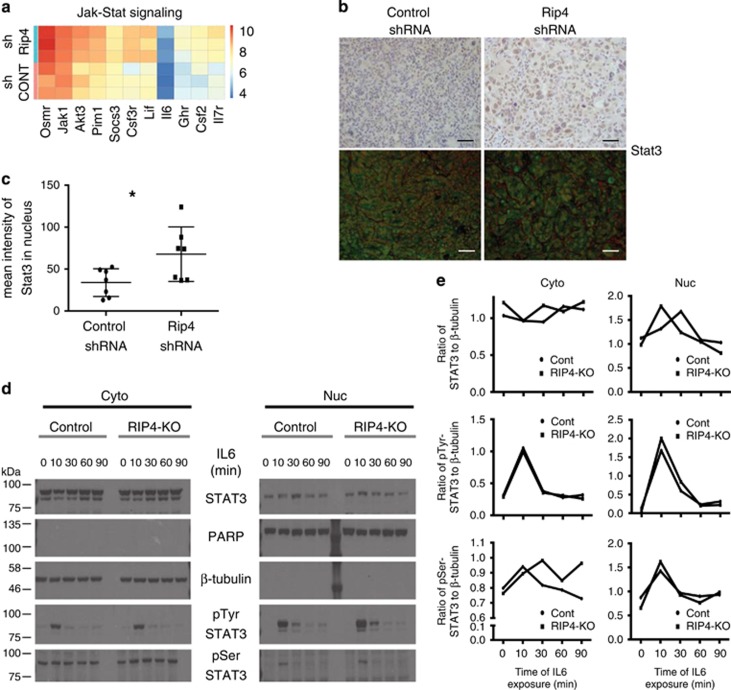
RIP4 inhibits IL6 signaling. (**a**) Heat map of Jak-Stat signaling genes differentially expressed in tumors with Rip4 knockdown compared to controls. (**b**) Representative IHC (top) and IF (bottom) images of paraffin-embedded lung tumor sections stained with Stat3. For IF red (Hoechst) shows nuclei, green Stat3. Scale bars represent 20 *μ*m. (**c**) Quantification of the nuclear intensity of Stat3 from IF images of (**b**). (**d**) Control and RIP4 knockout (RIP4-KO) H2009 cells were treated with IL6 for different time intervals (shown in minutes). Nuclear (Nuc)–cytoplasmic (Cyto) proteins were extracted and blotted. PARP and *β*-tubulin were examined as nuclear and cytoplasmic controls, respectively. (**e**) Intensity of STAT3, pTyr-STAT3 and pSer-STAT3 shown in (**d**) was quantified using ImageJ and normalized to that of PARP or *β*-tubulin

Gene expression profiling followed by gene set enrichment analysis (GSEA) of tumors with Rip4 knockdown highlighted activation of Jak-Stat signaling but no difference in NF-*κ*B (Figure 2a). IHC and immunofluorescence (IF) analysis of tumor sections with Rip4 knockdown showed increased phospho-serine 727 Stat3 (pSerStat3) and nuclear Stat3 levels (Figures 2b and c,Supplementary Figure S2a) but no difference in phospho-tyrosine 705 Stat3 (pTyrStat3) (Supplementary Figure S2a). These results prompted further investigation into a functional link between RIP4 and STAT3. To trigger STAT3 activation, we stimulated cells with the cytokine IL6 in some experiments, because it is a potent activator of this pathway. Altering RIP4 levels by shRNA or by overexpression of WT RIP4 or kinase-dead RIP4 (RIP4KD) did not affect proliferation of H2009 cells *in vitro* (Supplementary Figure S2b). However, shRNA-mediated RIP4 knockdown resulted in enhanced pSerSTAT3, but not pTyrSTAT3, in response to IL6 (Supplementary Figure S2c), and to an increased basal and IL6-stimulated nuclear STAT3 levels (Supplementary Figure S2d). To confirm that the altered nuclear STAT3 level is transcriptionally relevant, we monitored the levels of a *bonafide* STAT3 target, *SOCS3*. RIP4 knockdown resulted in increased IL6-stimulated *SOCS3* expression (Supplementary Figures S2e and f). To confirm the effect of RIP4 on STAT3 signaling, we generated RIP4 knockout (RIP4-KO) H2009 cells using CRISPR/Cas9 gene editing (Supplementary Figure S2g). RIP4 deletion resulted in faster nuclear import of STAT3 upon IL6 treatment (Figures 2d and e). Also, these cells had increased and sustained pSerSTAT3 as well as a minimally increased nuclear pTyrSTAT3 (Figures 2d and e). Furthermore, overexpression of RIP4 or RIP4KD reduced nuclear STAT3 even in the absence of stimulation (Supplementary Figures S2h and i). These results highlight an inhibitory role for RIP4 in STAT3 signaling. Interestingly, overexpression of STAT3 resulting in increased nuclear STAT3 was sufficient to recruit RIP4 to the nucleus suggesting the presence of an inhibitory feedback loop (Supplementary Figure S3a). To investigate if RIP4 alters STAT3 nuclear export, we treated RIP4 overexpressing H2009 cells with the nuclear export inhibitor leptomycin B and monitored nuclear STAT3 thereafter, by western blot or by quantification from IF microscopy. Leptomycin B treatment rescued RIP4-mediated reduction of nuclear STAT3 suggesting that RIP4 promotes its nuclear export (Supplementary Figures S3b–d). However, since RIP4 deletion results in faster nuclear import of STAT3 upon IL6 stimulation (Figures 2d and e), the inhibitory effect of RIP4 on IL6 signaling is probably not restricted to STAT3 nuclear export. Finally, although RIP4 is known to activate NF-*κ*B signaling, reducing NEMO did not alter the impact of RIP4 on IL6-mediated STAT3 nuclear localization (Supplementary Figure S3e). Altogether, these results demonstrate that RIP4 inhibits STAT3 signaling, partly by promoting its nuclear exit.

### RIP4 regulates expression of LOX

**Figure 3:**
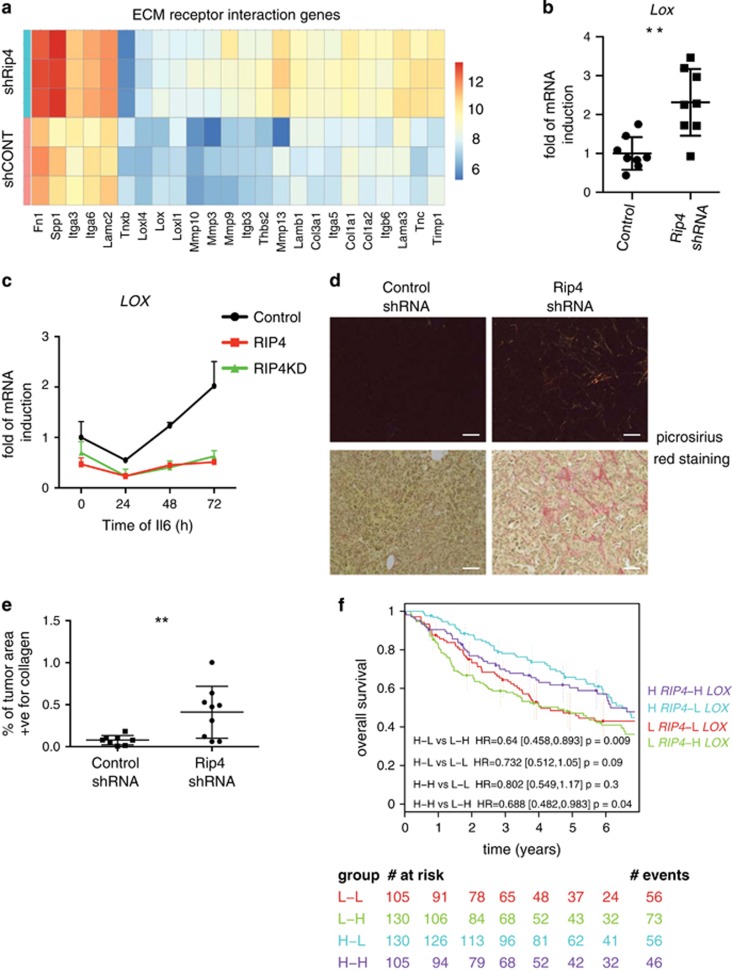
RIP4 counteracts IL6-mediated induction of the ECM remodeling *LOX*. (**a**) Heat map of ECM receptor interaction genes differentially expressed in tumors with Rip4 knockdown compared to controls. (**b**) Real-time PCR was performed on cDNA generated from tumor mRNA using probes for *Lox*. Data show mean±S.D. (*n*=8). Mann–Whitney test was used. **P<0.01. (**c**) H2009 cells expressing either RIP4 or RIP4KD were treated with IL6. Real-time PCR was performed on cDNA generated from mRNA using probes for *LOX*. Data show mean±S.D. (*n*=3). (**d**) Representative picrosirius red stained images of paraffin-embedded sections of control and Rip4 shRNA tumors taken using polarized light (top) and normal unpolarized light (bottom) are shown. Scale bars represent 50 *μ*m. (**e**) Quantification of collagen from images of (**d**) under polarized light. (**f**) Overall survival of patients with lung adenocarcinoma, divided into four groups based on *RIP4* and *LOX* expression, is represented as a Kaplan–Meier plot. Number of patients who have follow-up for each year and group has been indicated at the bottom. Cox proportional hazards regression model was used

Microarray analysis of tumors with shRip4 uncovered the up-regulation of ECM receptor interaction genes (Figure 3a). Lysyl oxidase (Lox), an enzyme involved in collagen crosslinking, has been linked to the formation of pre-metastatic niches at distant organs.^36^ Importantly, increased *Lox* expression was seen in tumors with shRip4 (Figure 3b). Extended (both 48 and 72 h) treatment of H2009 with the STAT3 activator IL6 elevated the levels of *LOX*, which was completely abolished upon overexpression of RIP4 or RIP4KD (Figure 3c). Probably because exposure of H2009 to IL6 reduces the levels of RIP4, no difference was detected in the amount of *LOX* induction in cells with shRIP4 (Supplementary Figures S4a and b). As collagen is the substrate of LOX, we monitored collagen deposition using picrosirius red staining of tumor sections, and quantified the signals under polarized light. This showed a significant increase in the amount of collagen in tumors with shRip4 (Figures 3d and e). Finally, we analyzed human lung AC samples for *LOX* expression, and determined that *LOX* alone was not a prognostic factor of survival (Supplementary Figure S4c). However, when considered together, high *RIP4* and low *LOX* levels in tumors were associated with good prognosis (Figure 3f). These results show that RIP4 inhibiting STAT3 prevents induction of LOX, an enzyme involved in ECM remodeling that contributes, with RIP4, to the prognosis of patients with lung AC.

### RIP4 reduces the invasiveness of lung cancer cells

**Figure 4:**
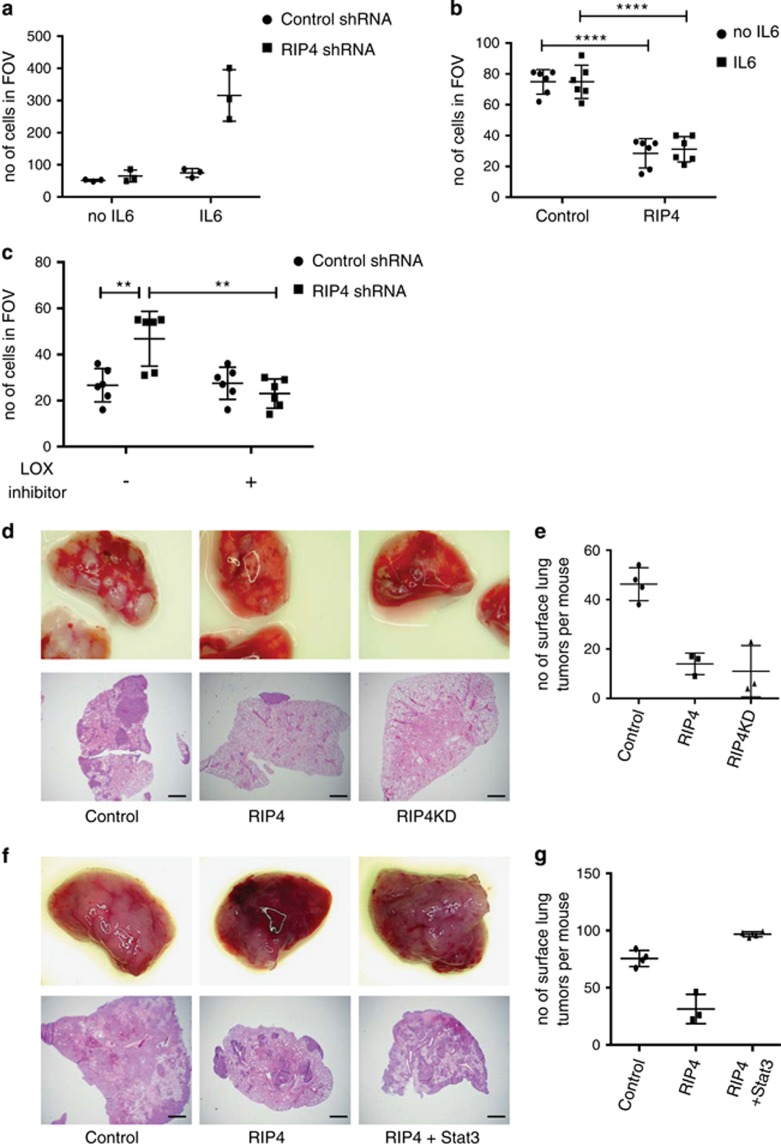
RIP4 reduces invasiveness of lung cancer cells. (**a**,**b**) Matrigel-collagen invasion assay was performed on H2009 cells with control or RIP4 shRNA (**a**) or RIP4 overexpressing cells (**b**). Cells were treated with IL6 for 24 h prior to the experiment. (**c**) Invasion assay was performed with IL6-treated H2009 cells expressing control or RIP4 shRNA. Cells were treated with the LOX inhibitor BAPN for 24 h. (**d**) M8 cells expressing doxycycline-inducible RIP4 or RIP4 KD were injected into the tail vein of 10-week old C57BL/6 mice. Control mice received M8 cell with inducible RIP4KD without doxycycline. Thirty one days after injection lungs were removed and pictures were taken (top). Paraffin-embedded lung sections were stained by H&E (bottom). Scale bars represent 500 *μ*m. (**e**) Number of visible tumors on the surface of the lungs was counted under a stereoscope. (**f**) M8 cells expressing doxycycline-inducible RIP4 or RIP4 and Stat3 were injected into the tail vein of 10-week old C57BL/6 mice. Control mice received M8 cell and doxycycline. Thirty days after injection lungs were removed and pictures were taken (top). Paraffin-embedded lung sections were stained by H&E (bottom). Scale bars represent 500 *μ*m. (**g**) Number of visible tumors on the surface of the lungs was counted under a stereoscope. For **b** and **c** student *t*-test was used. **P<0.01, ****P<0.0001. FOV, field of view; KD, kinase dead

**Figure 5:**
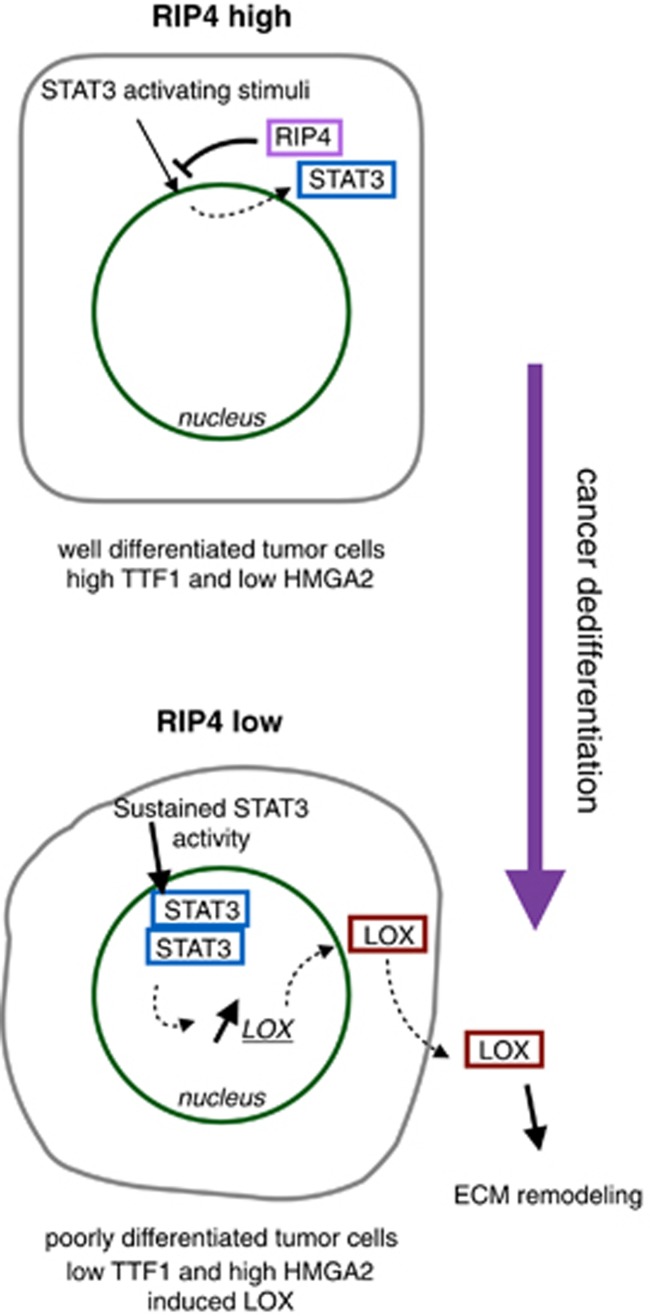
Model of the effect of RIP4 in tumor differentiation. RIP4 inhibits STAT3 activity, and maintains lung cancer cells in a well-differentiated epithelial state marked by high levels of TTF1 and no HMGA2. However, upon RIP4 knockdown, sustained signaling through STAT3 promotes the expression of ECM remodeling genes. This results in cancer cell dedifferentiation marked by expression of HMGA2 and low levels of TTF1

Next we investigated the role of RIP4 on cancer cell invasion both *in vitro* and *in vivo* using matrigel invasion assays and tail vein injections, respectively. Treating cells with IL6 for 24 h or knocking down RIP4 alone had no effect on cell invasiveness (Figure 4a). However, IL6 stimulation in RIP4 knockdown cells led to a drastic increase in invasion, suggesting that enhanced activation of STAT3 enabled by RIP4 knockdown is necessary to promote invasiveness (Figure 4a). Moreover, overexpression of RIP4 or RIP4KD reduced the invasiveness of the cells through collagen (Figure 4b, Supplementary Figure S5a). Treatment of H2009 cells with a LOX inhibitor (BAPN) attenuated the invasiveness mediated by IL6 stimulation+RIP4 knockdown (Figure 4c). Next, we developed a cell line, M8, from a KP mouse tumor, and stably expressed RIP4 (WT or KD) or a control vector. Confirming our results obtained in human cells, overexpression of RIP4 or RIP4KD reduced the *in vitro* invasiveness of M8 cells through collagen (Supplementary Figures S5b and c), without interfering with their growth (Supplementary Figure S5d). Importantly, tail vein injections revealed a significant reduction in the number of tumors developing in the lungs of mice injected with either RIP4 or RIP4KD-overexpressing cells compared to control cells (Figures 4d and e). This suggests that RIP4 reduces the invasive potential of the cancer cells, a crucial step for metastasis. Interestingly, 4 of the 6 tumors extracted from mice injected with RIP4 or RIP4KD-expressing cells had lost RIP4 expression (Supplementary Figure S5e), further supporting the notion that RIP4 reduces the metastatic potential of these cells. To confirm that the effect of RIP4 on invasiveness of lung cancer cells is due to its inhibitory effect on STAT3, we developed M8 cells that can overexpress RIP4 and/or Stat3. Overexpressed Stat3 partly localized to the nucleus in control and RIP4 overexpressing conditions (Supplementary Figure S5f). Here again, there was a reduced number of tumors formed in the lungs after injection of RIP4 overexpressing cells compared to control. More importantly, co-expression of Stat3 in these cells restored the number of lung tumors to that seen in control conditions (Figures 4f and g), revealing how the inhibitory action of RIP4 on STAT3 signaling impacts tumor cell invasiveness. Altogether, our results show that enhanced STAT3 activity achieved upon RIP4 down-regulation promotes the expression of ECM remodeling genes such as LOX, which contributes to cancer cell de-differentiation and metastasis (Figure 5).

## Discussion

Activation of STAT3 signaling has been implicated in the progression of multiple cancers,^37, 38^ and is known to contribute to oncogenic Kras-driven tumorigenesis.^38, 39^ Tumor cell-specific expression of mutant gp130 (membrane bound gp130^Y757F^ receptor which mediates Il6 signaling that can not be suppressed by Socs3) in a Kras mutant-driven mouse model of lung cancer resulted in increased tumor burden, which was reverted by the abrogation of hyper-activated Stat3 through either deletion of *Il6* or heterozygous deletion of *Stat3*.^10^ In this study, the contribution of Stat3 signaling was assessed up to 6 weeks after tumor initiation when the majority of lesions are atypical adenomatous hyperplasia or early ACs. However, the contribution of Stat3 signaling in late-stage Kras-mutant lung AC, frequently the stage at which most human patients are diagnosed, is still unclear.

STAT3 can be activated in different cell types and in response to multiple stimuli that include cytokines and growth factors. Therefore, the identification of modulators of STAT3 signaling could have therapeutic implications in various pathologies including cancer. Our work has identified RIP4 as a novel regulator of STAT3 signaling in lung tumors. Reduced RIP4 expression in human lung cancer cells enhanced STAT3 signaling whereas the opposite was true upon overexpression of RIP4, in a kinase activity-independent manner. Analysis of KP tumors with Rip4 knockdown showed increased nuclear Stat3 levels by IHC and an enrichment of Jak-Stat signaling by microarray. These tumors progressed to a poorly differentiated state as indicated by the differentiation markers Ttf1 and Hmga2. In human lung AC, *RIP4* levels were significantly reduced in poorly differentiated tumors, which is consistent with a recent finding reporting reduced RIP4 levels in poorly differentiated human tongue squamous cell carcinoma.^40^ Thus, the effect of RIP4 on tumor differentiation might not be restricted to lung AC.

A novel tumor suppressive role for Stat3 in Kras-driven lung AC was recently identified. Tumor cell-specific Stat3 knockout in Kras^G12D^ tumors resulted in increased tumorigenesis.^14^ Mechanistic analysis showed that cytoplasmic Stat3 retained NF-*κ*B in an inactive state. Despite increased tumorigenesis, Stat3-deficient tumors did not progress to a more invasive or metastatic state. Though inactive, cytoplasmic Stat3 might be tumor suppressive, the contribution of activated nuclear Stat3 needs to be further evaluated. A contrasting study reported that activated Stat3 promoted NF-*κ*B nuclear retention and hence tumorigenesis.^41^ Since RIP4 is known to activate NF-*κ*B in a kinase activity-dependent manner, we examined the activation of NF-*κ*B in tumors with Rip4 knockdown. Our results suggest that the contribution of Rip4 to lung tumor differentiation is NF-*κ*B independent.

The ability of cancer cells to extravasate and colonize distant organs is a crucial step in metastasis. Tail vein injection of cancer cells overexpressing RIP4 or RIP4KD significantly reduced the number of colonies found in the lung suggesting that RIP4 reduces the metastatic potential of the cells in a kinase-independent manner. This effect of RIP4 on invasiveness was rescued by a concomitant overexpression of Stat3. Overexpression of RIP4 or RIP4KD significantly reduced the migration of cells through matrigel and collagen, an important constituent of the basement membrane. Conversely, stimulation with IL6 potentiated the migration of cells with RIP4 knockdown suggesting that sustained STAT3 signaling promotes the metastatic potential of these cancer cells.

LOX, a secreted amine oxidase involved in collagen crosslinking, has been associated with invasion of cancer cells at the primary site^42, 43^ and also formation of pre-metastatic niches at distant sites.^44^ In Kras(G12D)-mediated oncogenesis, Lox has been reported to promote lung AC progression and invasion through excess collagen deposition and *β*1 integrin signaling.^45^ Inhibition of Lox enzymatic activity alleviates this effect. Microarray analysis of tumors with Rip4 knockdown showed an increased expression of various extracellular remodeling enzymes including *Lox*, *Loxl1* and *Loxl4*. Picrosirius red staining of these tumors demonstrated an increase in collagen deposition. A recent study on tumor initiating cells showed that pSerSTAT3 promoted collagen17 expression resulting in enhanced tumor initiating capacity of these cells.^46^ Our *in vitro* analyses showed that STAT3 pathway activation promoted the expression of *LOX*, which was reverted upon overexpression of RIP4, WT or KD. Altogether with the analysis of human lung AC samples that showed that patients with high *RIP4* and low *LOX* levels had the best prognosis, these results suggest that ECM remodeling participates in the progression of lung AC to a poorly differentiated malignant state observed upon loss of RIP4.

Mutant KRAS-driven cancers are challenging to treat due to the lack of efficient targeted therapy. Our study highlights the importance of sustained Stat3 signaling, and its regulation by Rip4, in an autochthonous model of oncogenic Kras-driven lung cancer. STAT3 signaling blockade using JAK inhibitors could prove to be therapeutically useful in late-stage lung AC. Besides differentiation of lung and tongue cancer, RIP4 has also been involved in developmental differentiation disorders such as bartsocas papas syndrome, features of which are recapitulated in *Rip4* knockout mice. Future studies evaluating the role of STAT3 signaling in malignancies or in such developmental disorders could prove to be therapeutically useful.

## Materials and Methods

### Cell culture and counting

NCI-H2009, human embryonic kidney (HEK) 293T and mouse KP (M8) cells were cultured in RPMI supplemented with 10% FBS and 1% penicillin–streptomycin (5000 U/ml). For serum-deprived conditions, cells were cultured in RPMI without FBS. When cells were 80–90% confluent, they were split using 2 ml of 0.05% trypsin and reseeded onto fresh plates. For doxycycline-inducible system, cells were cultured in RPMI supplemented with FBS, penicillin–streptomycin and 2 *μ*g/ml of doxycycline hyclate (D9891-1G; Sigma-Aldrich, Schnelldorf, Germany). For IL6 (CHI-HR-20006; Chimerigen Laboratories, Adipogen, Epalinges, Switzerland) treatment, stock concentration was prepared at 100 *μ*g/ml and later diluted in supplemented RPMI to 50 ng/ml final concentration used for stimulation. For leptomycin B (L2913-.5UG; Sigma-Aldrich) treatment, cells were cultured in RPMI with 10 ng/ml leptomycin B. To measure the number of live cells, 10 *μ*l of cells were mixed with 10 *μ*l trypan blue and counted using an automated cell counter (Countess, Life Technologies, Zug, Switzerland). LOX inhibitor 3-Aminopropionitrile fumarate salt (BAPN) was purchased from Sigma-Aldrich (A3134-5G).

### Real-time polymerase chain reaction

RNA was extracted from cells and tumors using trizol (15596018, ThermoFisher Scientific, Reinach, Switzerland). One microgram of RNA was used for reverse transcription using High capacity cDNA reverse transcription kit (4368814, ThermoFisher Scientific). Real-time PCR was done using Taqman universal PCR master mix (4324018, ThermoFisher Scientific) and Taqman probes for the studied genes (ThermoFisher Scientific). Levels of mRNA were normalized to *GAPDH* and *Rpl30* for human and mouse cells, respectively.

### Protein extraction and western blot

Cell proteins were extracted using RIPA buffer (20 mM Tris pH8.0, 50 mM NaCl, 0.5% sodium deoxycholate, 0.1% SDS, 1 mM Sodium orthovanadate, protein inhibitor cocktail (Sigma-Aldrich)). Cell lysates were treated with benzonase (70746, Merck Millipore, Schaffhausen, Switzerland). To obtain nuclear and cytoplasmic fractions, cells were lysed in EMSA-A buffer (20 mM Hepes pH 7.6, 20% glycerol, 10 mM NaCl, 1.5 mM MgCl_2_, 0.2 mM EDTA, 1 mM DTT, 0.1% NP-40, 1 mM sodium orthovanadate, protein inhibitor cocktail) and centrifuged. The supernatant was collected as the cytoplasmic fraction and the pellet was washed thrice with EMSA-A. The nuclear proteins were extracted by resuspending the pellet in EMSA-B (similar to EMSA-A with an exception of 500 mM NaCl). All extracted proteins were mixed with 3 × sample loading buffer (187.5 mM Tris-HCl, 0.03% Phenol Red, 6% SDS, 30% glycerol, 30 mM DTT). Samples were then run through 8% or 10% polyacrylamide gels for 35 min at 200 V. The proteins were then transferred to PVDF membranes. The membranes were blocked with 5% milk in PBS-tween followed by blotting with primary antibody. RIP4 (1:50, H00054101-M01 Abnova, LuBioScience, Luzern, Switzerland), STAT3 (1:1000, 4904 Cell Signaling, BioConcept AG, Allschwil, Switzerland), PARP (1:1000, 9532 Cell Signaling), *β*-tubulin (1:1000, sc-9104 Santa Cruz Technology, LabForce AG, Muttenz, Switzerland), phospho-tyrosine STAT3 (1:1000, 9145 Cell Signaling), phospho-serine STAT3 (1:1000, 9134 Cell Signaling), p65 (1:1000, sc-372 Santa Cruz Technology), p100/p52 (1:1000, 05-361 Merck Millipore) and NEMO (1:1000, sc-8330 Santa Cruz Technology) antibodies were used.

### Histology

Tumors and lungs were fixed in 1 : 10 volume ratio of 3.7% formaldehyde solution (A0823.0500, Axon Lab, Le Mont-sur-Lausanne, Switzerland) for at least 24 h and then embedded in paraffin. Sections of 4 *μ*m thickness were cut using a microtome and mounted on polarized glass slides (Super Frost Plus, Thermo Scientific). The sections were stained with H&E for morphologic assessment.

### Histopathology

Histopathologic analysis of tumors was performed in a blinded fashion by a board certified veterinary pathologist. The parameters considered to evaluate morphologic differentiation of AC were: overall architecture of the mass and cells arrangement patterns, cells shape, anisocytosis, anisokaryosis, number of nuclei/presence of multinucleated giant neoplastic cells, cell and nuclear polarity.

### Immunohistochemistry and immunofluorescence

Antigen retrieval for IHC was done on tissue embedded polarized glass slides with 10 mM sodium-citrate and blocked with 0.3% hydrogen peroxide (Sigma-Aldrich). Tissue sections were then blocked with goat serum and stained with primary antibody. Hmga2 (1 : 400, 8179 Cell Signaling), Ttf1 (1 : 200, 5883-1 Epitomics, Burlingame, CA, USA), Stat3 (1 : 800), phospho-tyrosine Stat3 (1 : 400), phospho-serine Stat3 (1 : 400), p65 (1 : 200, sc-372 Santa Cruz Technology), phospho-histone H3 (1 : 200, 9701 Cell Signaling) and cleaved caspase-3 (1 : 200, 9664 Cell Signaling) antibodies were used. Biotinylated secondary antibody was used followed by treatment with avidin bound peroxidase enzyme (ABC kit, Vectastain, Reactolab SA, Servion, Switzerland). DAB and hydrogen peroxide were used as substrate for the peroxidase enzyme. Harris haematoxylin was used as counter stain. Cells were fixed using 10% formaldehyde for IF. Stat3 (1 : 800) antibody was used for staining. The nucleus was stained using Hoechst 33342. Quantification of IF was performed using ImageJ. Clipping mask for the nucleus was created using the signal from Hoechst 33342. This mask was then applied on the corresponding Stat3 image to quantify the nuclear Stat3 intensity. A clipping mask for the whole cell was created using Stat3 signal. Subtracting the nuclear mask from the whole cell mask created a cytoplasmic mask. This was then applied on the Stat3 image to quantify the cytoplasmic Stat3 intensity.

### Tail vein injections

Transduced M8 Cells were treated with doxycycline for 2 days before injection to overexpress RIP4 or RIP4KD. C57BL/6 mice receiving RIP4 or RIP4KD-overexpressing cells were given doxycycline in their food starting from 1day before injection until the end of the experiment. Control mice were injected with cells (not treated with doxycycline) and the mice did not receive doxycycline. A total of 2 × 10^5^ cells were injected through the tail vein. Lungs were extracted 31 days after injection and embedded in paraffin.

### Autochthonous mouse model

Transduction units (3500) of lentivirus carrying plasmid with Cre recombinase was delivered through the trachea of 13-week old *Kras*^*LSLG12D/wt*^*; p53*^*fl/fl*^ mice. Littermates were infected with virus carrying shp53 or shRip4. Tumor growth was monitored using *μ*CT. Mice were sacrificed 18.5 weeks after tumor initiation. Lungs and tumors were either used for mRNA extraction or embedded in paraffin. All mouse experiments were performed with the permission of the Veterinary Authority of the Canton de Vaud, Switzerland (license number VD2391).

### Plasmid and viral production

Flag-tagged human RIP4 and RIP4KD constructs in pCR3.1 plasmids were described previously.^47^ To transduce H2009 cells, inducible human RIP4 and RIP4KD constructs were cloned into pCW22 lentiviral vector using the forward oligo 5′-CTCGTT AACGCCACCATGGATTACAAAGACGATGACGATAAAATGGAGGGCGACGGCGGG-3′ and the reverse oligo 5′-CTCTTAATTAACTAGGTCTTGCTTCGCCG-3′. The PCR products were digested and cloned using Hpa1 and Pac1. pLKO.1 plasmid expressing shRNA against human RIP4 was purchased from Sigma-Aldrich. shRNA against mouse Rip4 was designed using pSICOLIGOMAKER 1.5 program prepared by annealing the forward oligo 5′- TGAAGCCATTTGCAGATGAATTCAAGAGATTCATCTGCAAATGGCTTCTTTTTTC-3′ and reverse oligo 5′-TCGAGAAAAAAGAAGCCATTTGCAGATGAATCTCTTGAATTCATCTGCAAATGGCTTCA-3′. They were cloned into U6-shRNA-Pgk-Cre plasmid from Professor Tyler Jacks’ Laboratory (MIT, Cambridge, USA) using XhoI and HpaI. 293 T cells were transfected using lipofectamine 2000 (ThermoFisher Scientific) with the necessary lentiviral vector mentioned earlier and lentiviral packaging vectors pMD2G (VSV-G protein) and pCMVR8.74 (obtained from Professor Didier Trono’s Laboratory, EPFL, Lausanne, Switzerland). After 6–8 h transfection, the medium was replaced with FBS-supplemented RPMI. The conditioned medium-containing virus was harvested 36 and 60 h post transfection. To infect cell lines, conditioned medium was added to the cells for 48 h and then cells were selected using puromycin or blasticidin. To infect mice, the virus was concentrated by ultra-centrifugation (50 000 × *g*, 120 min, 16 °C). The pellet was then solubilized in PBS. The TRE-mouseStat3-HA plasmid was from a previously described library.^48^

### CRISPR/Cas9 gene editing

RIP4 knockout H2009 cells were generated using CRISPR/Cas9 gene editing. Lentiviral plasmid pL-CRISPR.EFS.tRFP was used to transduce H2009 cells to develop control (without sgRNA) or RIP4 knockout cells (with sgRNA against RIP4). The following sgRNAs were used:

sgRNA1 forward: 5′ CACCGCGTGAACTCGCCCGCGTCGA 3′

sgRNA1 Reverse: 5′ AAACTCGACGCGGGCGAGTTCACGC 3′

sgRNA2 forward: 5′ CACCGCAGGCTGGGCGAGCACTTGA 3′

sgRNA2 reverse: 5′ AAACTCAAGTGCTCGCCCAGCCTGC 3′

The oligonucleotides were annealed and ligated into the plasmid, which was digested using Bsmb I restriction enzyme. Lentiviral vectors were produced using these plasmids. Following transduction, H2009 cells were amplified and sorted for RFP. RFP-positive cells were amplified and used for experimentation.

### Matrigel-collagen invasion assay

Collagen type I (354236, Corning, Amsterdam, Netherlands) solution was prepared in PBS/NaOH with a final NaOH concentration of 20 mM and Collagen concentration of 3 mg/ml. In total, 75 *μ*l growth factor-reduced Matrigel (356231, Corning), 100 *μ*l non-supplemented DMEM, 200 *μ*l collagen solution and 125 *μ*l cell suspension containing 62 500 cells were mixed using vortex. A volume of 150 *μ*l gel-cell mixture was added to Millicell-PCF 8.0 *μ*m insert of 12 mm diameter and put into 24 well plates. A volume of 300 *μ*l supplemented medium was added to the bottom and 650 *μ*l was added to the top of the chamber. After incubation for 16 h at 37 °C, the chambers were removed and the medium was aspirated. The membrane at the bottom of the transwell was washed with PBS and fixed with cold methanol. The cells were stained with Hoechst 33342 and counted under the microscope.

### Bioinformatics

All statistical analyses were performed using the free high-level interpreted statistical language R (version 3.0.1) and various Bioconductor packages (http://www.Bioconductor.org).

#### (A) Exploration of public data sets:

1. A collection of five public lung cancer transcriptome resources has been used to investigate *RIP4* and *LOX* expression levels and their impact on patient survival. Four data sets were collected from the multi-site study described by Shedden *et al.,* ^49^ and the fifth data set is described in the study by Zhu *et al.* ^50^ The five resources were combined as follows. Raw expression data from Affymetrix Human Genome U133A Array were downloaded from Gene Expression Omnibus (GEO: http://www.ncbi.nlm.nih.gov/geo/). Each data set was background subtracted and normalized using the robust multi-array average (RMA) method from affy package (version 1.44.0). The batch effect correction method ComBat from sva package (version 3.12.0) was applied to combine the data sets. Patients were divided into high and low risk groups on the basis of *RIP4* and *LOX* expression; continuous expression variable was dichotomized using the median. A survival analysis was performed with the Kaplan–Meier method to compute survival curve, hazard-ratio and confidence interval. Differences between curves were assessed using the log rank test (Mantel–Cox). Distributions of expression values have been examined for different grade of tumor differentiation with the non-parametric Kruskal–Wallis rank-sum test. The *P*-values issued from the test are reported on the figures. All tests were two-sided and *P*-values below 0.05 were considered statistically significant.
2. TCGA's Lung Adenocarcinoma (LUAD) was retrieved from The Cancer Genome Atlas (TCGA project: http://cancergenome.nih.gov/). Samples with both clinical information and mRNA (RNASEQV2) data were selected and the data was downloaded using the RTCGA package.^51^ Normalized RSEM gene counts as provided by TCGA were taken. As a filtering step, only genes with counts per million>1 in at least 50 samples are kept for further analysis and then counts are converted to log-counts per million using voom function from limma package.^52^ Out of 480 samples, we had survival information for 448 samples; mutational information was available only in 198 samples. A total of 73 samples with a mutation in *KRAS* (G12 or Q61), 99 samples with a mutation in *TP53* (nonsense, missense, frame shift mutations, in frame deletion) and survival data were used for survival analysis. Differences in expression levels between mutant samples groups were assessed by Kruskal–Wallis rank-sum test.

#### (B) Microarray analysis

RNA was prepared and hybridized to Affymetrix GeneChIP 2.0 ST arrays (Affymetrix, Santa Clara, CA, GPL16570) by the Lausanne Genomic Technologies Facility, Center for Integrative Genomics (University of Lausanne, Switzerland). Data were normalized using the RMA algorithm from the xps package (version 1.26.1) and analyzed using the limma package (version 3.22.7). Differential expression of genes between controls and Rip4 KO mice has been assessed with the standard procedure described in the limma package using the *eBayes* method. The GSEA has been performed with the *roast* method of the limma package, a self-contained rotation gene set test proposed by Wu *et al.*^53^ Gene sets were collected from MSigDB (version 4) with homolog mapping from human to mouse genes. A false discovery ratio threshold of 0.05 was applied for calling a gene set as statistically significantly associated with the observed gene expression difference between the conditions.

### Transfection

H2009 cells were grown to 60% confluence in a 10 cm plate. Control siRNA, Rip4 siRNA and KRAS siRNA were purchased from ThermoFisher Scientific. siRNAs were diluted to a final concentration of 10 nM. Lipofectamine RNAiMAX (ThermoFisher Scientific) was mixed with siRNA in non-supplemented medium and added to cells. After 8 h, the medium was replaced with FBS-supplemented RPMI medium.

### Accession numbers

The accession number for the Microarray Data is GEO: GSE81154.

### Statistics

Cell culture experiments were repeated at least in biological triplicates to accommodate technical variations. If the positive and negative control conditions did not meet the experimental criteria, the samples were excluded from analysis. For mouse experiments, initial analysis of groups of five or more mice for each condition suggested a requirement and sufficiency of three mice per group unless an abnormal variation within the cohort was observed. During the course of experimentation, the mice were regularly scanned for tumors using *μ*CT. If the mouse was found to have deteriorated health (as suggested by the veterinarian), abnormal for the tumor burden and age, the mouse was excluded from analysis. All *μ*CT scans were performed blind. All animals in a particular experiment were age matched. However, the selection of animals in each group was entirely random. The statistical methods applied are mentioned in the figure legends. For samples obtained from mouse experiments, Mann–Whitney test was appropriate for the sample size and analysis.
