# Supplementary figures and images for: RIP4 inhibits STAT3 signaling to sustain lung adenocarcinoma differentiation
Source: Cell Death Differ. 2017 Jun 2;24(10):1761–71. doi: 10.1038/cdd.2017.81 (PMC5596425; doi:10.1038/cdd.2017.81)

**a**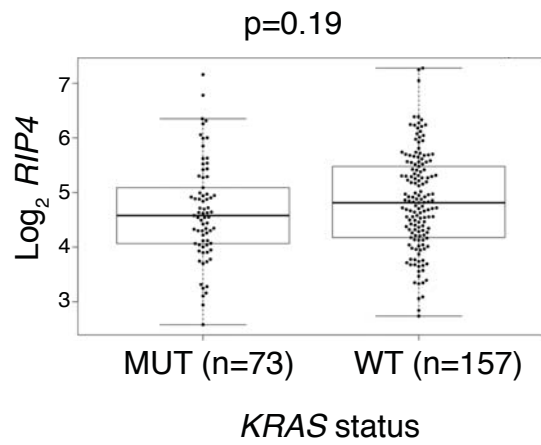**b**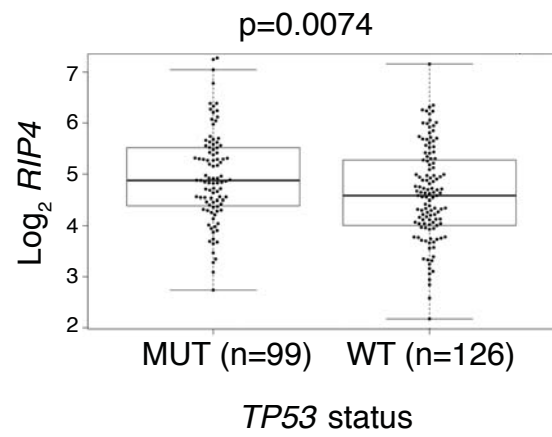**c**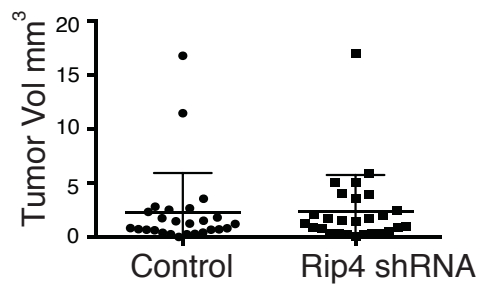**e**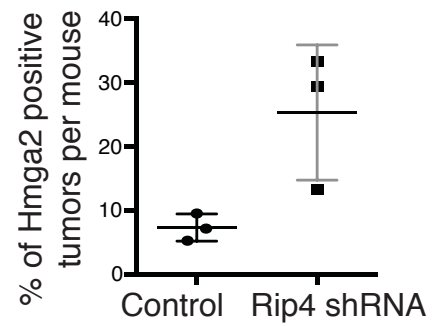**d**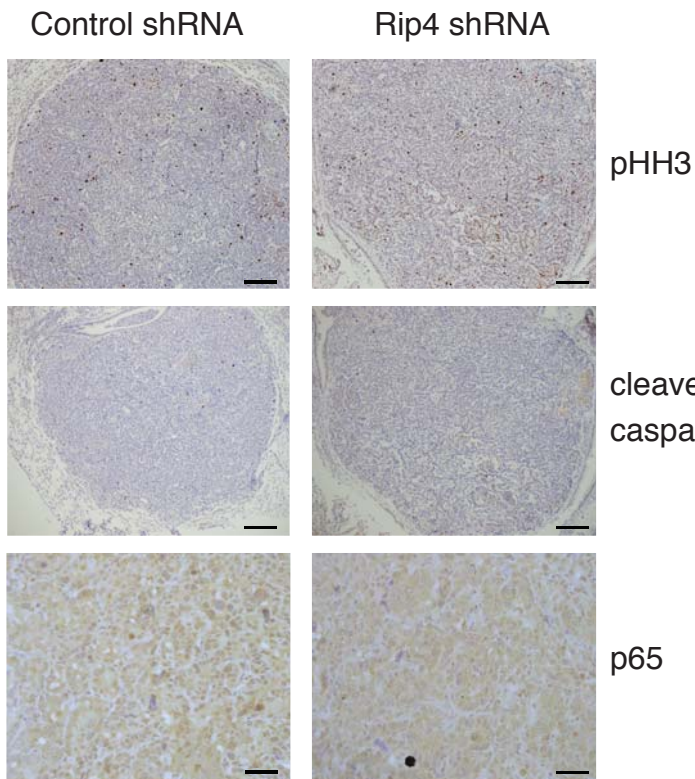**f**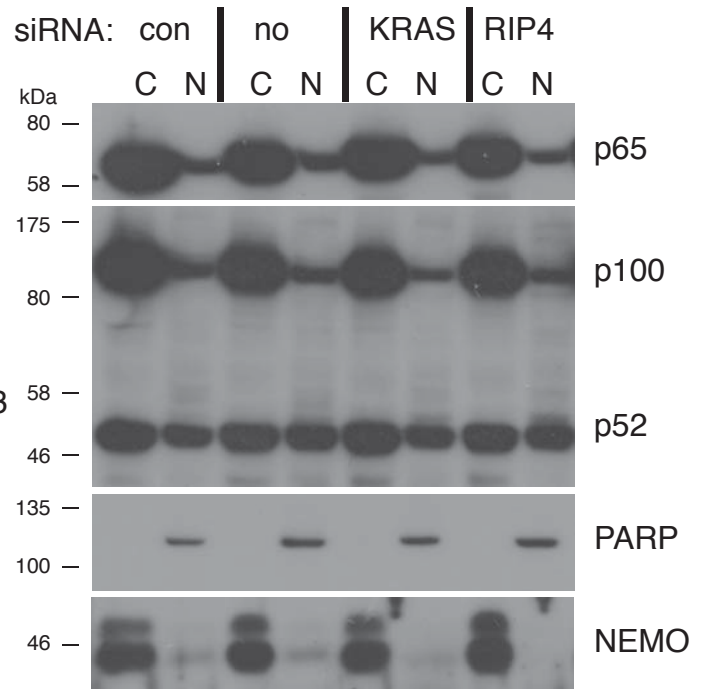

Supplement: Supplementary Figure 1 [file cdd201781x1.pdf]

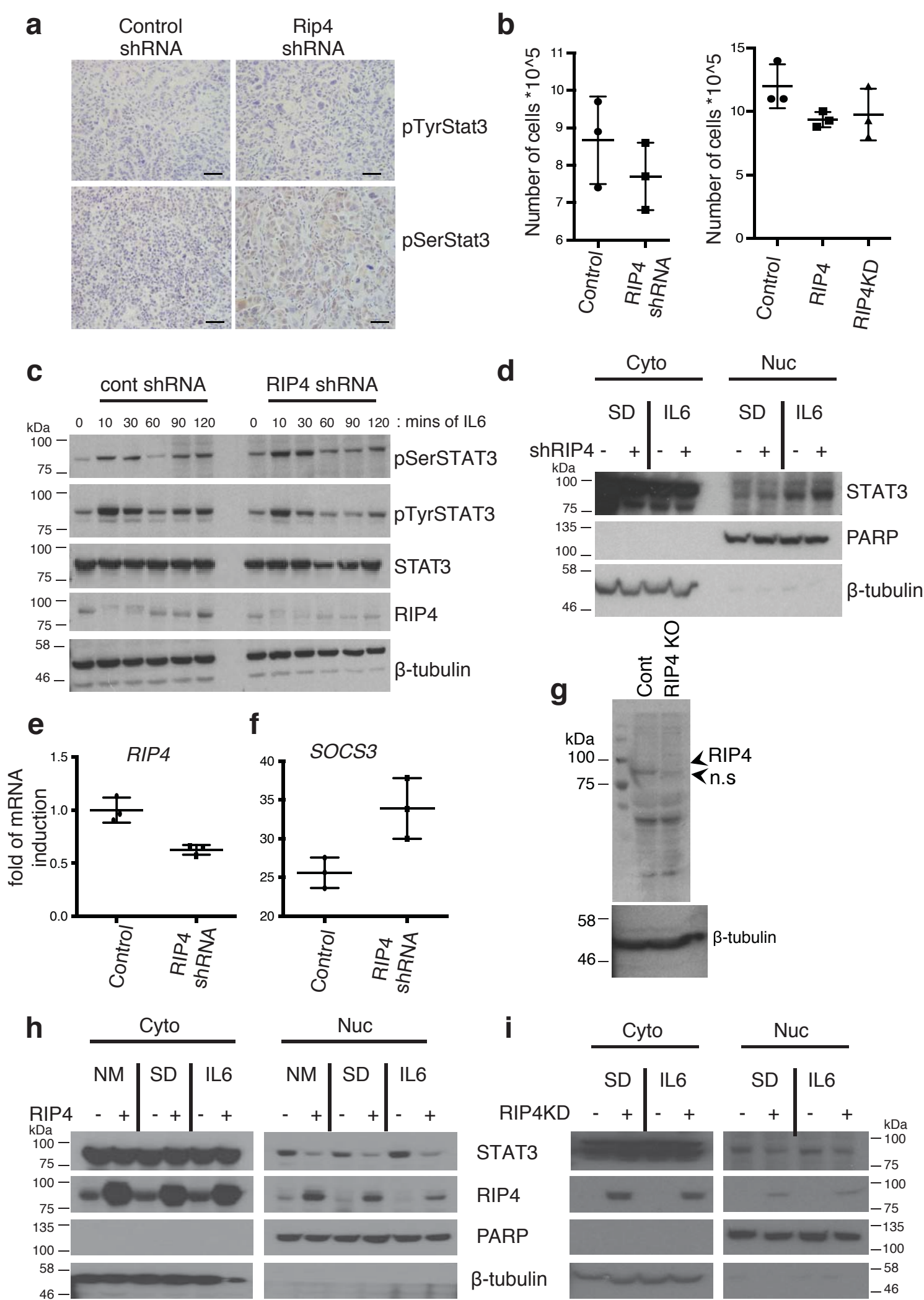

Supplement: Supplementary Figure 2 [file cdd201781x2.pdf]

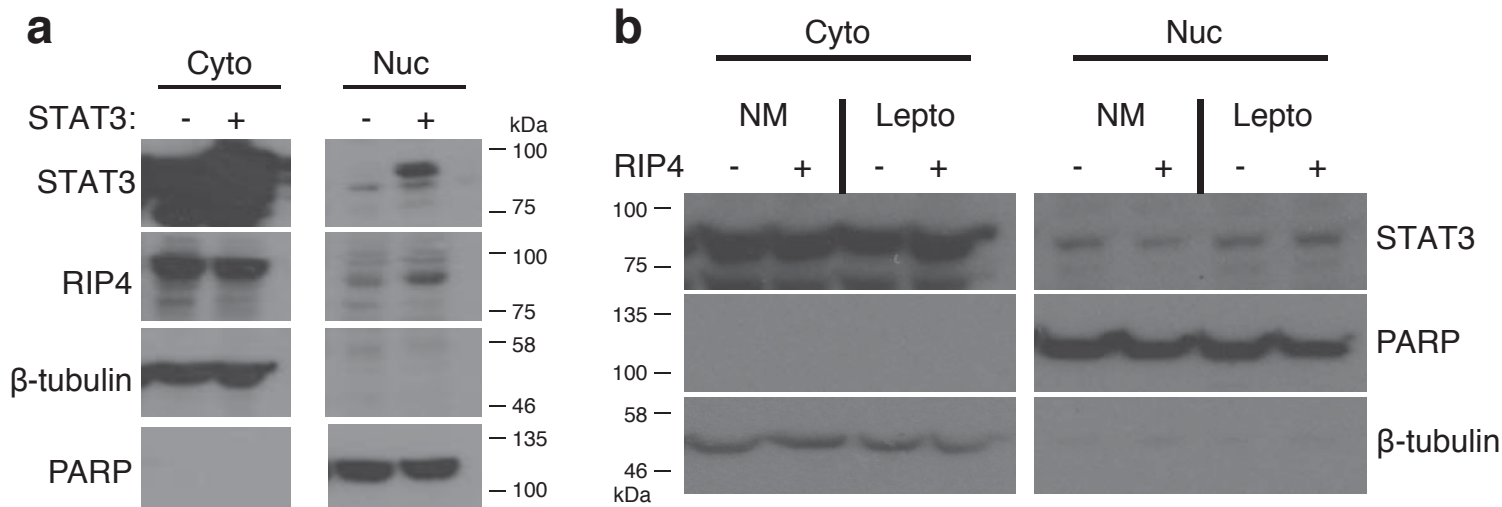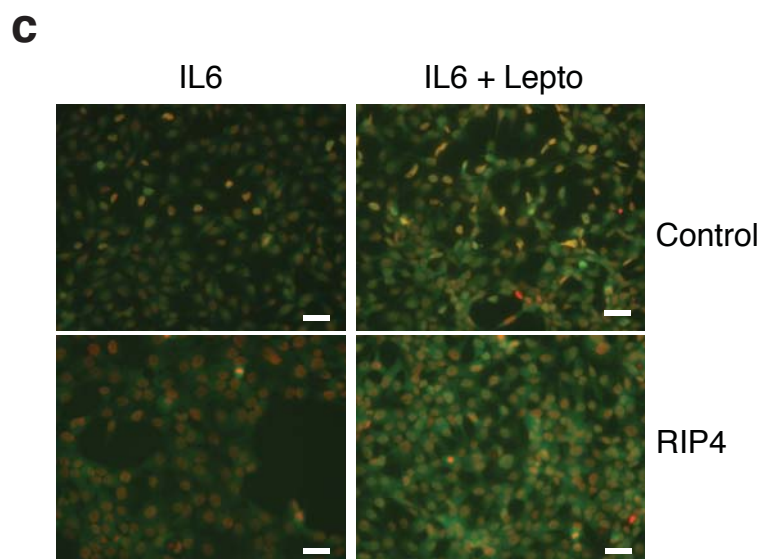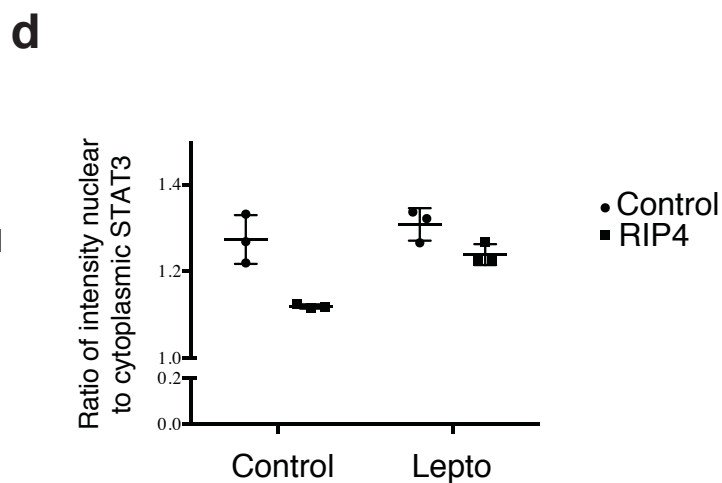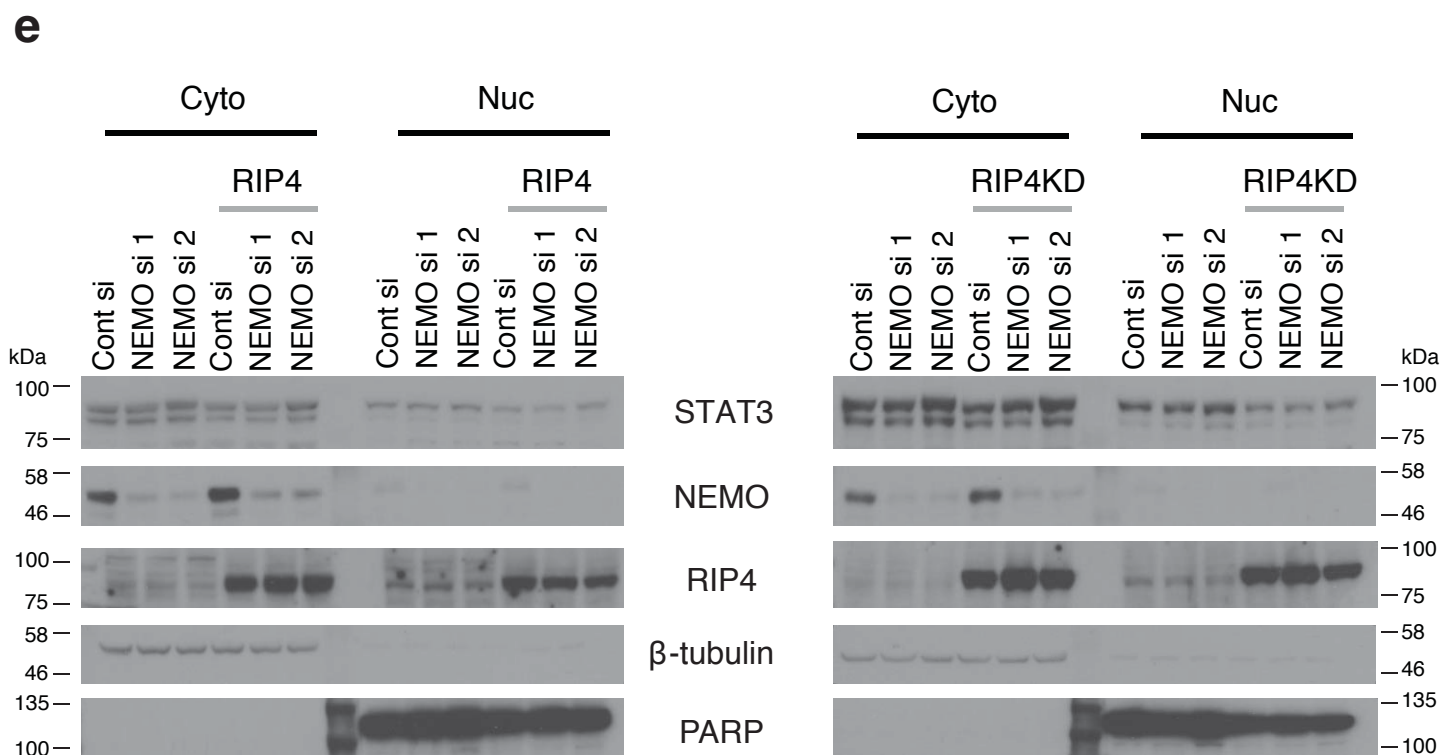

Supplement: Supplementary Figure 3 [file cdd201781x3.pdf]

**a**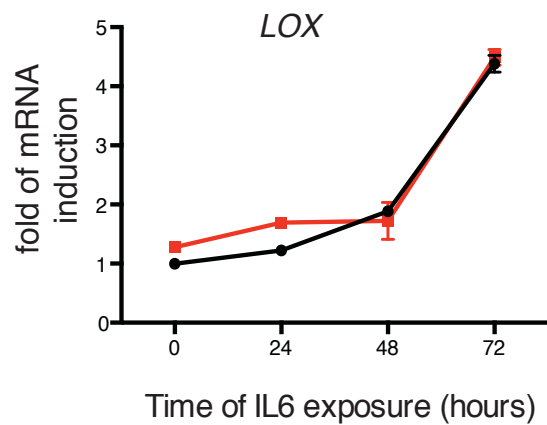**b**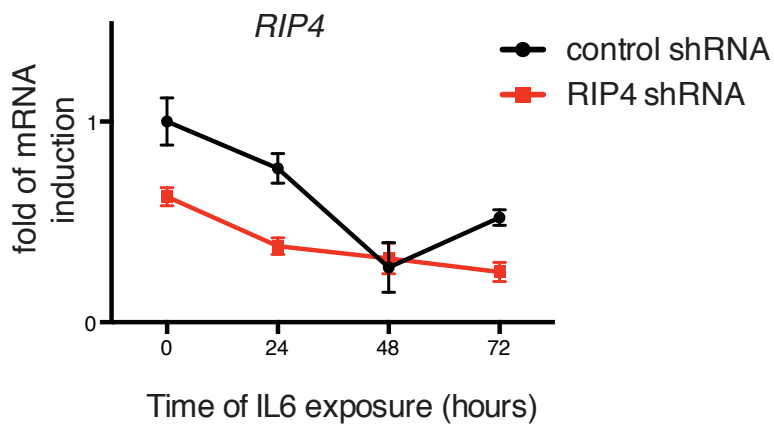**c**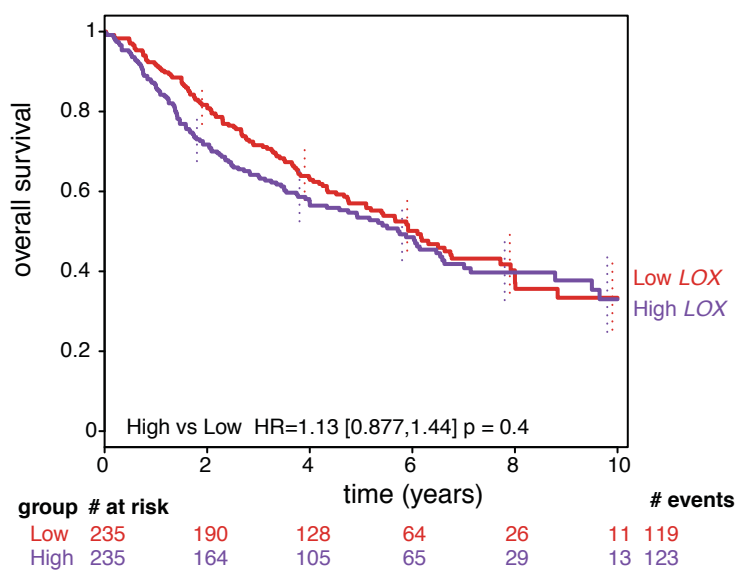

Supplement: Supplementary Figure 4 [file cdd201781x4.pdf]

**a**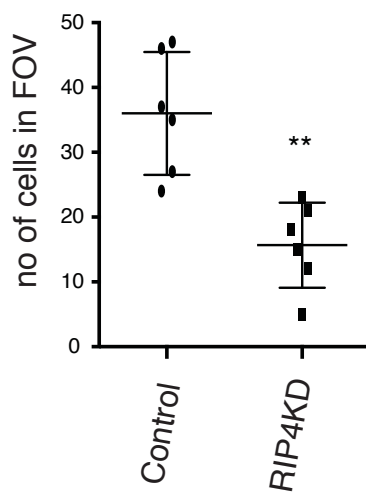**b**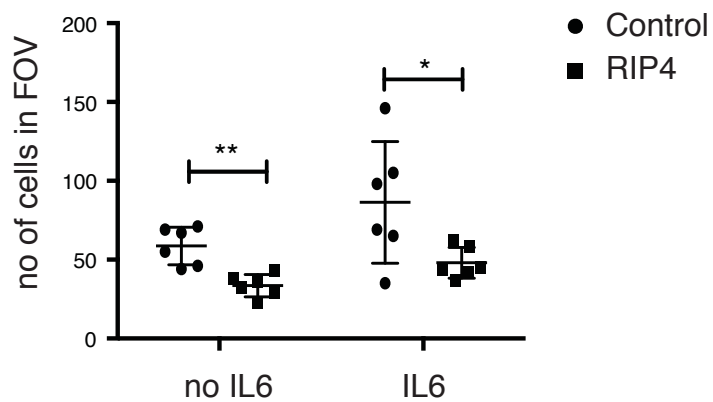**c**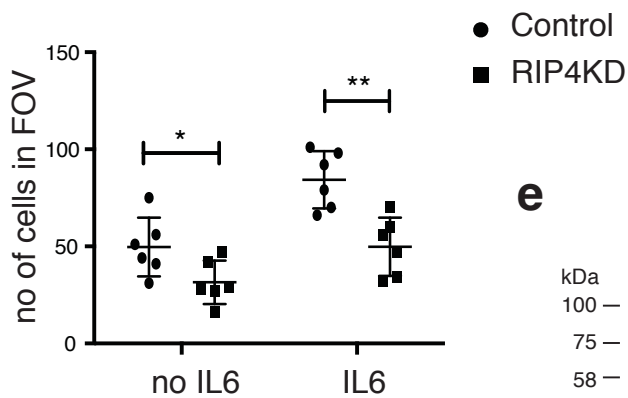**e**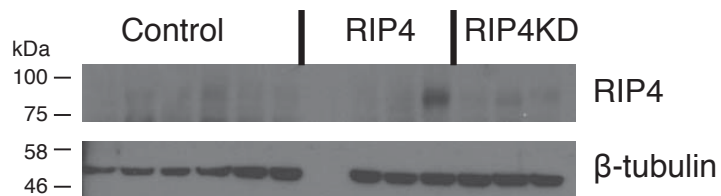**d**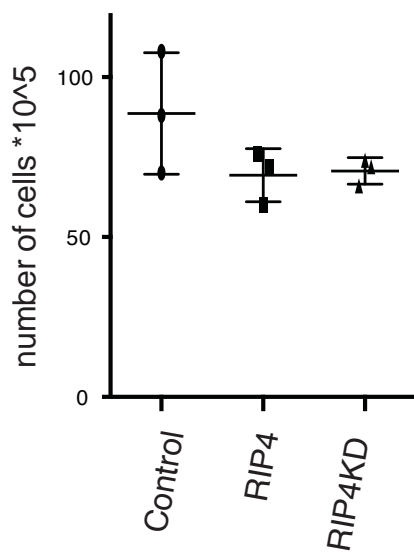**f**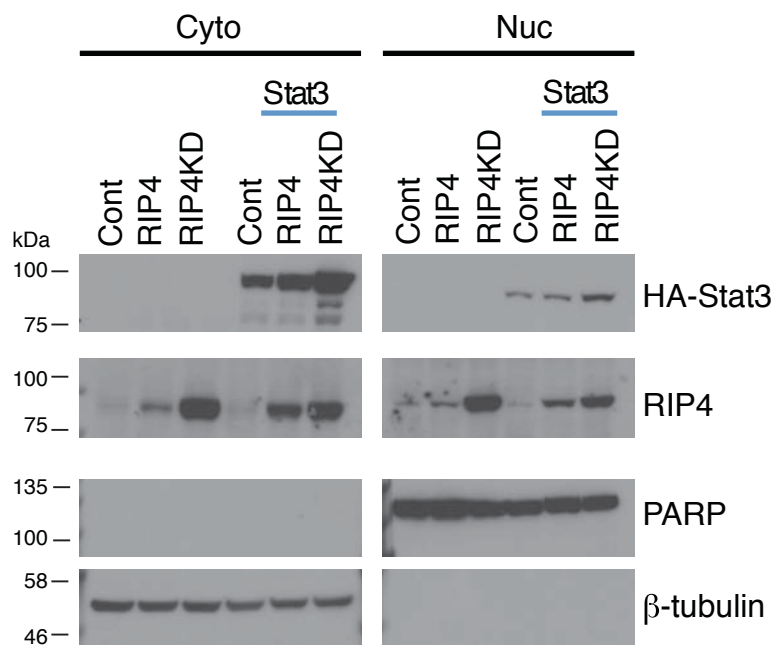

Supplement: Supplementary Figure 5 [file cdd201781x5.pdf]
