# Supplementary material for: RIP4 inhibits STAT3 signaling to sustain lung adenocarcinoma differentiation
Source: Cell Death Differ. 2017 Jun 2;24(10):1761–71. doi: 10.1038/cdd.2017.81 (PMC5596425; doi:10.1038/cdd.2017.81)
Supplement: Supplementary Legends [file cdd201781x6.pdf]

Supplementary Figure 1: **Effect of Rip4 on tumor proliferation and NF-κB**

(**a**) *RIP4* mRNA levels in human lung adenocarcinoma samples with or without *KRAS* mutation are plotted. (**b**) *RIP4* mRNA levels in human lung adenocarcinoma samples with or without *TP53* mutation are plotted. (**c**) Tumor volumes at 18.5 weeks post infection, from mice infected with control shRNA (n=26) or Rip4 shRNA (n=27), were measured using μCT. Data shown as mean +/- sd. Mann-Whitney test showed no statistical difference between the tumor volumes. (**d**) Representative images of paraffin embedded sections stained for p65, phospho-histone H3 (pHH3) and cleaved caspase-3 are shown. Scale bar for p65 is 20 μm. Scale bars for pHH3 and cleaved caspase-3 are 50 μm. (**e**) Paraffin embedded sections were stained for Hmga2 and the number of Hmga2 positive lesions were calculated in each mice (n=3 per group). Data are shown as mean +/- sd. (**f**) Nuclear (Nuc)-cytoplasmic (Cyto) proteins were extracted from H2009 cells treated with control, KRAS, RIP4 or no siRNA. Western blot was performed to monitor p65, p100 and p52. PARP and NEMO are controls for Nuc and Cyto fractions, respectively.

Supplementary Figure 2: **Effect of RIP4 on STAT3 signaling**

(**a**) Representative images of paraffin embedded lung tumor sections stained with pTyr705-Stat3 and pSer727-Stat3. Scale bars represent 20 μm. (**b**) Cell proliferation was measured for H2009 cells expressing RIP4 shRNA, RIP4 or RIP4KD. Cell numbers were measured using Trypan Blue 5 days after plating 2*10^5 cells. (**c**) Western blots of proteins extracted from IL6 treated H2009 cells expressing control or RIP4 shRNA. β-tubulin levels are shown as controls. (**d**) H2009 cells expressing either control shRNA or RIP4 shRNA were treated with IL6. Nuclear (Nuc) – cytoplasmic (Cyto) proteins were extracted and blotted. PARP and β-tubulin were examined as nuclear and cytoplasmic controls, respectively. (**e**) *RIP4* levels are shown by real-time PCR in H2009 cells with control or RIP4 shRNA. Mean+/-sd (n=3). (**f**)

H2009 cells with control or RIP4 shRNA were treated with IL6 for 40 min. *SOCS3* levels analyzed by real-time PCR were normalized to *GAPDH* and represented as fold induction compared to unstimulated conditions. Data show mean +/- sd (n=3). (**g**) Proteins were extracted from control and RIP4 knockout (RIP4-KO) cells and blotted. RIP4 level was examined. β-tubulin was used as control. (**h**) H2009 parental cells or stable RIP4 expressing cells were grown in normal culture conditions (NM) or serum deprived for 12 h (SD) or treated with IL6 for 30 min after 12 h of serum deprivation (IL6). Nuclear (Nuc) and cytoplasmic (Cyto) proteins were extracted followed by western blot. PARP and β-tubulin were examined as nuclear and cytoplasmic controls, respectively. (**i**) H2009 cells expressing doxycycline inducible RIP4 kinase dead (RIP4KD) were treated and analyzed as in (**h**).

Supplementary Figure 3: **Effect of RIP4 on STAT3 nuclear localization**

(**a**) Nuclear (Nuc) – cytoplasmic (Cyto) proteins were extracted from H2009 cells with doxycycline inducible STAT3 expression and blotted. (**b**) Nuclear (Nuc)-cytoplasmic (Cyto) proteins were extracted from H2009 cells either overexpressing RIP4 or not. All cells were treated with IL6 for 3 h. Indicated conditions were treated with leptomycin B for 16 h. PARP and β-tubulin are nuclear and cytoplasmic controls, respectively. (**c**) Immunofluorescence was performed on H2009 cells either overexpressing RIP4 or not. All cells were treated with IL6 for 30 minutes. Indicated conditions were treated with leptomycin B for 16 h. STAT3 is shown in green and nuclei in red (Hoechst). Scale bar represents 20 μm. (**d**) Intensity of nuclear and cytoplasmic STAT3 shown in (**c**) was quantified using ImageJ and represented as a ratio. 3 different fields of view were used for each condition. (**e**) H2009 cells expressing either RIP4 or RIP4 kinase dead (RIP4KD) were treated with IL6 for 30 minutes. Cells were transfected with control siRNA or either of the two different siRNAs against NEMO for 48 h

prior stimulation. Nuclear (Nuc) – cytoplasmic (Cyto) proteins were extracted and blotted. PARP and β-tubulin were examined as nuclear and cytoplasmic controls, respectively.

Supplementary Figure 4: **Effect of RIP4 on LOX**

(**a**, **b**) H2009 cells expressing either control or RIP4 shRNA were treated with 50 ng/ml IL6. Real-time PCR was performed on cDNA generated from mRNA using probes for *LOX* (**a**) or *RIP4* (**b**). Data show mean+/- sd (n=3). (**c**) Overall survival of patients with lung adenocarcinoma, divided into two groups based on median *LOX* expression (high and low), is represented as a Kaplan-Meier plot. Number of patients who have follow-up for each year and group has been indicated at the bottom. Cox proportional hazards regression model, p=0.4, *HR* hazard ratio=1.13.

Supplementary Figure 5: **Impact of RIP4 overexpression on invasiveness**

(**a**) Matrigel-collagen invasion assay was performed on H2009 cells expressing RIP4 kinase dead (KD). FOV, field of view. (**b**) Matrigel-collagen invasion assay was performed on M8 cells expressing RIP4. FOV, field of view. (**c**) Matrigel-collagen invasion assay was performed on M8 cells expressing RIP4 kinase dead (KD). FOV, field of view. (**d**) Cell proliferation was measured for M8 cells expressing RIP4 or RIP4KD. Cells were measured using Trypan Blue 5 days after plating 2*10^5 cells. (**e**) Proteins were extracted from 31 days old tumors derived from lungs of mice injected with M8 cells. Control tumors (n=3 for each RIP4 and RIP4KD) did not receive doxycycline. RIP4 and RIP4KD (n=3) are tumors extracted from mice treated with doxycycline. RIP4 levels are shown by western blot and β-tubulin was used as control. (**f**) Nuclear (nuc) and Cytoplasmic (cyto) proteins were extracted from M8 cells overexpressing HA-Stat3 and/or RIP4 and blotted for HA tag and RIP4. PARP

and β-tubulin are nuclear and cytoplasmic controls, respectively. For (**a**-**c**) student t-test was used. * $p<0.05$, ** $p<0.01$.
